# The Watershed as A Conceptual Framework for the Study of Environmental and Human Health

**DOI:** 10.4137/EHI.S1925

**Published:** 2009-02-18

**Authors:** Alan S. Kolok, Cheryl L. Beseler, Xun-Hong Chen, Patrick J. Shea

**Affiliations:** 1 Department of Environmental, Agricultural and Occupational Health, 987850 Nebraska Medical Center, Omaha, NE 68198; 2 Department of Biology, University of Nebraska at Omaha, 6001 Dodge Street, Omaha, NE 68182; 3 Department of Epidemiology, 987850 Nebraska Medical Center Omaha, NE 68198; 4 School of Natural Resources, 3310 Holdrege St., University of Nebraska-Lincoln, Lincoln, NE 68583-0996

**Keywords:** watershed, agrichemicals, environmental health, epidemiology, agricultural runoff, hormone disrupting chemicals

## Abstract

The watershed provides a physical basis for establishing linkages between aquatic contaminants, environmental health and human health. Current attempts to establish such linkages are limited by environmental and epidemiological constraints. Environmental limitations include difficulties in characterizing the temporal and spatial dynamics of agricultural runoff, in fully understanding the degradation and metabolism of these compounds in the environment, and in understanding complex mixtures. Epidemiological limitations include difficulties associated with the organization of risk factor data and uncertainty about which measurable endpoints are most appropriate for an agricultural setting. Nevertheless, it is our contention that an adoption of the watershed concept can alleviate some of these difficulties. From an environmental perspective, the watershed concept helps identify differences in land use and application of agrichemicals at a level of resolution relevant to human health outcomes. From an epidemiological perspective, the watershed concept places data into a construct with environmental relevance. In this perspectives paper, we discuss how the watershed can provide a conceptual framework for studies in environmental and human health.

## Introduction

When considering adverse human health outcomes in communities engaged in agriculture, drinking water is a key route of exposure. While application of pesticides to the land, or administration of pharmaceutical compounds to livestock, can lead to pesticide and hormone residues in drinking water and adverse human health outcomes, the relationship involves subtle yet complex interactions. For example, the relationship between land application of pesticides and surface water is influenced by precipitation and evapotranspiration, infiltration, ground water recharge and irrigation, runoff and surface water irrigation.^1^

Establishing ties between environmental health and human health is not only thwarted by the complexity of environmental interactions, but can be difficult given the current organization of human demographic and risk factor data. Generally these data are aggregated into established geographic census units, such as counties, census tracts, census blocks and census block groups. Contaminants, however, have no respect for census boundaries resulting in heterogeneity of exposure when the unit of analysis overlaps regions with differing geological characteristics. To identify significant associations between exposures and human health, within-group exposure must be homogenous. Determining the appropriate geographic census unit becomes a major issue when investigating human health outcomes because estimating the rate of disease in a population requires a denominator that represents the population at risk of the disease. Individuals residing in different watersheds or those residing in different regions of the same watershed may not have equivalent opportunities for exposure.

We propose that the watershed provides a valuable conceptual framework for studies focusing on the interaction between aquatic contaminants and environmental and human health. A watershed is the area of land where all of the water under it or draining off of it goes to the same place and includes both surface and ground water. Consequently, the environmental history of two individuals living some distance from each other but in the same watershed may be more closely related than that of two individuals living near each other but in different watersheds. From the perspective of human and environmental health, the relationship between watershed geography and contaminant distribution is critical and needs further exploration.

## The Elkhorn River Watershed

### Land use and surface water

In this article we will use the Elkhorn River watershed as a case study. The Elkhorn watershed, approximately 18,135 km^2^, is located in northeastern and north central Nebraska, encompassing parts of 24 counties. The dominant surface water feature is the Elkhorn River. The surface gradient within the watershed is modest, ranging from 606 m at O’Neill in the northwest to 366 m at Fremont in the southeastern corner, despite the fact that these two points are separated by over 245 river km. Rainfall also varies modestly from east to west, from an annual average of 75.9 cm (38.6 cm during the growing season) at Fremont to 59.4 cm (30.2 cm) at O’Neill.

**Figure 1.**
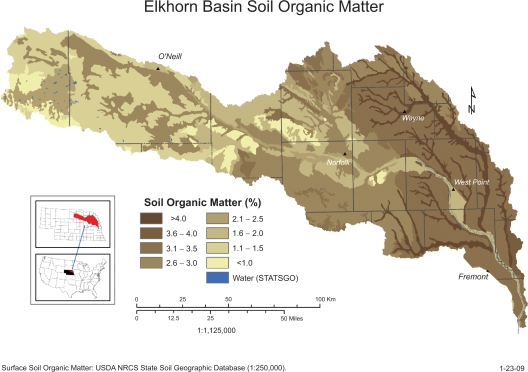
Soil organic matter in the Elkhorn River watershed.

**Figure 2.**
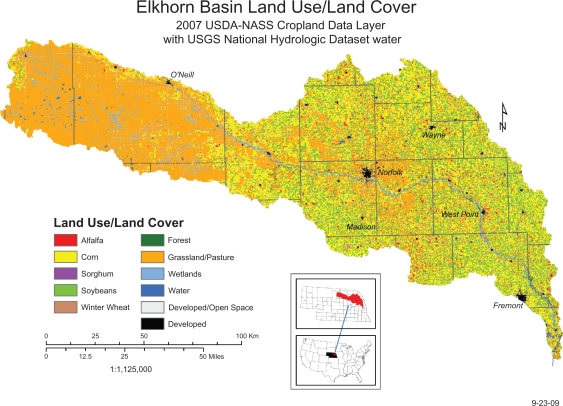
Land use in the Elkhorn River watershed.

**Figure 3.**
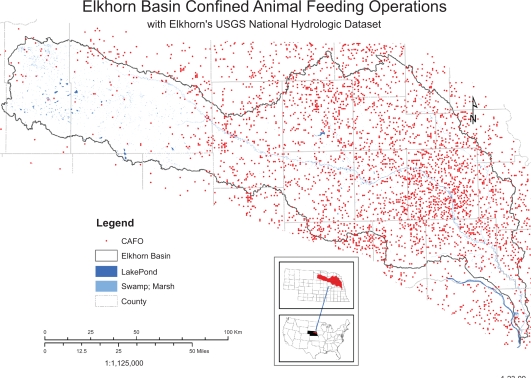
Confined beef cattle feeding operations in the Elkhorn River watershed.

While changes in elevation and annual precipitation are modest across the watershed, differences in soil type and agricultural practices are more pronounced. In the eastern portion of the watershed, silt and loess predominate whereas in the western portion of the watershed sandhills and shale predominate. These differences are also reflected in soil organic content, which is lower in the western portion of the watershed than in the east (Fig. 1). Corn and soybeans are the major row crops in the east, with a gradual change to wheat, pasture and rangeland further west in the watershed (Fig. 2). There are also differences in livestock practices, as cattle feedlots predominate in the east, whereas cow-calf operations predominate in the west (Fig. 3). Because the nature of agrichemicals used varies with land use in the watershed, the types and quantities of contaminants present in ground and surface waters will change from east to west.

The prevalence of row crops and beef cattle within the Elkhorn River watershed is in sharp contrast to the scant human population in the region. Excluding the two urban counties at the southeast corner of the watershed, the human population within the remaining 22 counties is approximately 232,000 and not all of these people live within the watershed. To put this number into perspective, there are about the same number of beef cattle in feedlots in one county within the watershed (Cuming County) as people in all 22 counties. Agricultural pesticides and veterinary pharmaceuticals would be expected to comprise a greater source of contamination than the waste stream from the communities (human pharmaceuticals, personal care products, cleaning products, industrial byproducts, etc) within the watershed.

### Ground water as a source of drinking water

Beneath the Elkhorn River watershed lies the Ogallala aquifer, one of the largest aquifers in the world. The Elkhorn River and the underlying ground water are connected; surface water reaches the ground water through infiltration, whereas ground water returns to the surface through wells and discharges to the Elkhorn River and its tributaries as baseflow. According to Chen et al.^2^ groundwater seepage through the streambed of the Elkhorn River near Neligh was as high as 0.94 m^3^/d per square meter at some locations, indicating that the Elkhorn River receives a large quantity of groundwater from the surrounding aquifers. In the hyporheic zone, the inflow from the stream to the streambed was also observed. The infiltration rate was up to 0.38 m^3^/d per square meter. There were 12,441 registered ground water wells within the watershed in 2005.^3^ Irrigation is the largest consumer of ground water, with approximately 1,100,000 acres supplied by approximately 8,400 wells in 2005.

People living in the Elkhorn River watershed get their drinking water from wells. The Nebraska Department of Natural Resources Wells Database lists 2812 registered domestic wells and 389 registered wells for public water supply systems in the Elkhorn River watershed. Importantly, all of the public water supply wells are located close to rivers or creeks and 266 wells are within 100 m of those waterways. The depth of these supply wells ranges from 10 to 135 m. The depth of about one third of these wells is less than 30 m, while another 33% are between 30–60 m. Only 15 wells are more than 100 m deep. While it is likely that agrichemical contaminants in groundwater may be more closely related to human health than those in surface water, groundwater in both the upper and lower Elkhorn River watershed occurs in alluvial aquifers that are often highly permeable and hydrologically connected to the rivers. For example, test-hole logs drilled near Pilger indicate that the Quaternary alluvial materials on both sides of the Elkhorn River consist mainly of sand and gravel.^4^ Electrical conductivity logs and sediment cores show that the sediments beneath the river channel near Pilger consist mainly of sand and gravel as well. Computer simulations for permeable alluvial aquifers, a hypothetical case by Chen^5^ and a case study by Abdel-Fattah,^6^ show that pumping in near-river wells can induce infiltration of river water into the streambed and if the pumping time is sufficiently long, the infiltrated river water will arrive at the pumping well. If the river contains contaminants, they may be carried to the hyporheic zone (the zone in which surface water mixes with ground water) and then into the water supply system.

### Geographic data and environmental health

Residents living in the Elkhorn River watershed are likely to be exposed to different agrichemicals depending upon their location. While local variations in the environment (water movement or management practices of individual farmers and ranchers) undoubtedly influence local water quality, we contend that the change from grassland to row crop agriculture is the dominant geographical issue of importance to environmental and human health in this region of Nebraska. Furthermore, dividing the watershed into two regions based upon land use (grassland vs. corn/soybean rotation) may be epidemiologically important, as the resultant sub-populations should be large enough for meaningful study. While we are not aware of any epidemiological studies that have focused on geographical variation within the Elkhorn River watershed, this may be a fruitful area for further study.

## Chemical Contaminants and the Elkhorn River Watershed

In an agricultural environment with a low human population density, such as the Elkhorn River watershed, pesticides used in row crop agricuture and growth-promoting steroids used by the beef cattle industry, may represent the greatest contribution of organic contaminants to the surface water. Recent research has shown that the biological effects of many of these compounds challenge traditional thinking about how contaminants behave in the environment. For example, compounds acting as endocrine disruptors may exhibit non-monotonic dose-response relationships, and may have biological effects at very low concentrations.^7^ These compounds may also disrupt developmental and reproductive processes, and their occurrence in drinking water may have direct, though subtle, human health consequences.

### The contaminants

#### Pesticides

Pesticide use within the Elkhorn River watershed is greatest in the eastern half of the watershed where corn and soybeans predominate. Historically, the herbicides atrazine and cyanazine (*s*-triazines), and alachlor and metolachlor (chloroacetanilides) were most widely used on these crops, with preference gradually shifting to a product containing a mixture of atrazine and acetochlor in corn. Pendimethalin or trifluralin (dinitroaniline herbicides), metribuzin (*as*-triazine), as well as alachlor, metolachlor and other herbicides have been used in soybeans. For many years dicamba (a substituted benzoic acid) and 2,4-D (a chlorophenoxyacetic acid applied in salt or ester form) have been widely used for postemergence broadleaf weed control in corn and remain in general use.

In recent years, the use of 2,4-D and dicamba in agriculture has been declining, in part due to the advent of low application rate herbicides such as nicosulfuron, primisulfuron, rimsulfuron and chlorimuron (sulfonylureas), mesotrione (benzocyclohexanedione), and cloransulam (sulfonanilide), which can be used for postemergence weed control in corn and soybeans. Another major change is the increasingly wide spread use of Roundup Ready^®^ (herbicide resistant) corn and soybeans, permitting the use of glyphosate (*N*-phosphonomethyl glycine) as the primary chemical weed control agent. For some time, the organophosphate insecticide chlorpyrifos has been widely used in both corn and soybean, along with terbufos and methyl parathion (also organophosphates), carbofuran and carbaryl (carbamates), and several other chemicals. More recently, insecticide use also has shifted to synthetic pyrethroids such as permethrin, esfenvalerate, cypermethrin, bifenthrin, cyfluthrin and cyhalothrin.

#### Growth-promoting compounds

As with pesticides, the use of growth-promoting steroids predominates in the eastern half of the Elkhorn River watershed. The predominant livestock in the Elkhorn River watershed are beef cattle; however animal operations are generally segregated from west to east. Cow-calf grazing operations predominate in the western portions of the watershed, and growth-promoting implants are routinely used on the slower growing calves. In contrast, animals held in the feedlots (heifers, fast-growing calves or steers) all receive growth-promoting implants.

For beef cattle held in feedlots, growth-promoting compounds are administered in feed or as a pelleted ear implant.^8^ There are currently six compounds listed for use as growth-promoting agents in beef cattle: trenbolone acetate, estradiol, testosterone, melengestrol acetate, progesterone, and zeranol. While single growth-promoting compounds can be administered, the most responsive implant for steers is a 5:1 to 10:1 ratio of trenbolone acetate and estradiol. Melengestrol acetate is given to heifers as a feed additive to prevent estrus, thereby channeling reproductive energy into somatic growth.

### The potential for off-site movement

When considering the potential for off-site movement of pesticides or growth-promoting compounds, some physiochemical properties of the compound are particularly important. Among these, the most important properties are rate of degradation (indicated by half-life) and affinity for soil (indicated by the organic carbon partition coefficient or K_oc_). With the exception of extremely soluble or insoluble pesticides, water solubility is less critical because field application rates typically result in soil solution concentrations well below the water solubility of the pesticide. The potential for agrichemical runoff in surface water is generally greatest when the K_oc_ is between 50 and 5,000 (leaching may predominate at K_oc_ < 50) and increases with persistence (longer half-life).

Pesticides are generally applied to agricultural fields as parent compounds, and much of the runoff contains parent compounds rather than metabolites. Some pesticides such as the triazine (e.g. atrazine) and chloracetanilide herbicides (e.g. alachlor, metolachlor and acetochlor), readily dissolve and move with water. Other compounds, such as the dinitroaniline herbicides (e.g. trifluralin and pendimethalin) and organophosphate insecticides (e.g. chlorpyrifos), more strongly associate with soil particles and organic matter and are transported primarily with eroded soil, particularly during times of high runoff from precipitation or irrigation.^9^

Unlike pesticides, which are applied to fields in parent form, growth-promoting compounds are deposited into the environment in both parent form and various metabolites.^8^ Growth-promoting compounds only enter the environment after passing through a beef heifer or steer. With the exception of melengestrol, the compounds are excreted primarily as water-soluble metabolites and conjugates. The primary route of excretion of androgens, estrogens and progestrogens is fecal, and fecal pats from steers implanted with a trenbolone:estradiol combination implant have been shown to contain androgenic steroids. All of the registered steroids are fairly lipid soluble; however, the metabolites are much more water soluble and as such more mobile.

### Sorption to soils

Agrichemicals must be bioavailable to be of concern to environmental or human health. Bioavailability is altered by sorption to soils. All agrichemicals have some affinity for organic matter and organic matter content is one of the most important factor determining adsorption and availability in soil.^10^ In the Elkhorn River watershed, soil organic matter increases from west to east. As a result, agrichemicals in runoff from croplands or animal operations in the eastern part of the watershed are likely to adsorb to those organically rich soils to a greater extent than to the organically poor soils of the west (Fig. 1). This greater affinity of the soils for agrichemicals in the eastern portion of the watershed may decrease their overall bioavailability.

### Land use and agrichemical residues in waters from the Elkhorn River

Pesticides occur in detectable concentrations throughout the Elkhorn River watershed.in stream water samples. Frenzel et al.^11^ reported that alachlor, atrazine, cyanazine, and metolachlor, were most commonly applied and detected (≥78% of stream water samples) for corn, sorghum, and soybean production in the Central Nebraska Basins Study Unit, a 30,000 square mile area of intensive agriculture extending from the Elkhorn River in the northeast south to the Platter River and including the Lincoln metropolitan area. Atrazine was detected in all stream samples. Other notable detections included the herbicides prometon (69% of stream samples), simazine (64% of samples), pendimethalin (37% of samples), propachlor (32% of samples), metribuzin (25% of samples) and trifluralin (20% of samples), along with the insecticides chlorpyrifos (24% of samples) and carbofuran (22% of samples). Concentrations in the water were found to depend upon seasonal application and rainfall patterns as the greatest concentrations were inevitably found during the growing season following intense rainfall shortly after herbicide application.

Relationships between proximity to feedlots and the occurrence and activity of steroidogenic compounds in the Elkhorn Riker have not been well established. For example, Soto et al.^12^ analyzed water samples from six sites throughout the lower Elkhorn River (Nebraska) for estrogenic activity (E-screen), androgenic activity (A-screen) and the occurrence of estrone, 17-β-estradiol, 17-α-trenbolone, 17-β-trenbolone and trendione. Estrogenic activity was found at all six sites, with the greatest activity in a feedlot retention basin, and at the confluence of the retention pond drainage ditch and the Elkhorn River (approximately 0.5 km from the retention pond). Estrone, a metabolite of 17-β-estradiol was detected at each of the six sites but did not account for much (3%–46%) of the estrogenic activity. With respect to androgenic activity, Soto et al.^12^ found androgenic activity at all sites, with the highest activity at the retention basin and lowest at the control site. Androgenic compounds were detected only at marginal levels.

In a follow-up study on the Elkhorn River, Kolok et al.^13^ attempted to correlate the concentrations of estrone, 17-β-estradiol, 17-α-trenbolone, progesterone and melengestrol acetate to the proximity to beef cattle CAFOs (confined animal feeding operations). Passive samplers were deployed at four sites; two in small creeks immediately downstream from major CAFO operations, one deployed in the mainstream Elkhorn River, immediately downstream from the Norfolk wastewater treatment plant, and a fourth at a reference site. No clear-cut relationships were discernable between location within the watershed and amount of steroids collected in the passive samplers. Additional research is needed to clarify relationships between deployment sites and the occurrence of these compounds in surface waters.

### Mixtures

Chemical mixtures may result from application of multiple pesticides to agricultural fields or administration of multiple pharmaceuticals to livestock. They also result from the commingling of runoff from fields sprayed with different compounds, or runoff from a CAFO commingling with runoff from agricultural fields. Understanding agrichemical mixtures is important when discerning impacts on human and environmental health. For example, Belden et al.^14^ showed that the most common pesticide mixture found in streams was acetochlor-metolachlor, followed by alachlor-atrazine-metolachlor or alachlor-atrazine-metolachlor-cyanazine. Atrazine and metolachlor have been shown to induce CYP19 (aromatase), thereby potentially promoting the conversion of androgens to estrogens resulting in higher levels of 17-β-estradiol in exposed human populations.^15^,^16^ Likewise, the estrogen metabolite estrone has been detected at all sites sampled throughout the Elkhorn River watershed. Interactions between metabolite of steroids and pesticides are currently unknown. Nevertheless, the occurrence of agrichemical mixtures, particularly in streams, implies that the combined toxicity of pesticides in aquatic ecosystems as well as health and environmental impacts may be greater than that of any single pesticide present.

Agrichemical degradation products and metabolites may pose a problem with respect to the overall level of contamination of a watershed. Most metabolites are less toxic than the parent compound, although some degradation products such as desethylatrazine (DEA) and metolachlor ESA (ethanesulfonic acid) and metabolites such as estrone or 17-α or -β trenbolone are active agents that pose similar or different risks to human health and (or) the environment. Mixtures of parent compounds and their metabolites need to be considered when assessing potential impacts on human and environmental health. This is a daunting but essential task.

## Sentinel Markers of Exposure to Hormone Disrupting Chemicals in Humans

A number of studies in the past decade have suggested that agrichemicals may have multiple effects on human health, including impaired reproductive capacity, altered immune and thyroid function, and cancer risk. One mechanism by which these agrichemicals, can elicit adverse health effects is via their action as hormone disrupting chemicals (HDCs). From a human health perspective, it may be particularly important in agriculturally dominated systems to have surveillance endpoints that will be useful in evaluating the effect and impact of HDCs on the human population. The remainder of this perspective recommends a few such endpoints.

### Sex ratios

Sex ratios can be calculated from readily available birth data. They may be sensitive indicators of environmental hormonal effects in cross-sectional analysis comparing regions in a watershed that are vulnerable to drinking water contaminants to less vulnerable areas. Even slight alterations in this ratio over time would indicate that further study is warranted as the ratio is stable and well-characterized in human populations. Although no mechanism has been shown to link HDCs to changes in the sex ratio, there are a number of ways in which it could occur and studies suggest that it does. HDCs may alter the ratio of testosterone to human chorionic gonadotropin in men or they might affect DNA methylation patterns, as has been shown in mouse embryos.^17^

Experimental aquatic and mammalian models demonstrate changes in sex ratios when exposed to HDCs. For example, a municipal sewage treatment plant in southern Finland treats waste from about 1 million residents.^18^ The effluent contained measurable estrogenic steroids and nonylphenol derivatives. In samples of 100 or 150 zebrafish, estrogenic municipal effluents altered the sex ratio of three generations of continuously exposed zebrafish to favor females.^18^ Sex ratio changes in humans were also observed immediately following the 2,4,5-trichlorophenol explosion in Seveso, Italy, in 1976, with a gradual recovery in the years since the accident.^19^ Calculating the male to female sex ratio in babies born in California between 1960 and 1996 revealed no alterations in the sex ratio and investigators concluded that the apparent changes noted in other studies were likely due to confounding by changes in demographic factors.^20^ However, another possible explanation exists for the apparent lack of consistency across studies. HDCs may differentially affect sex ratios in exposed men compared to exposed women and this difference is not reflected in the population-based California study. The measure may best be utilized when both parents are exposed and compared to parents not exposed based on being in a region of higher risk, such as what occurred in Seveso, Italy.^21^ As an environmental indicator of HDC exposure, the sex ratio might be more appropriately applied within the context of a watershed comparing the western region of the Elkhorn River watershed over a number of years during which there has been an increase in agrichemical use and the number and size of CAFOs to the eastern region with different exposures. We have not yet explored this marker and are just beginning to understand the underlying biology. As this is an easy and quick calculation to make with data that are readily available, further efforts should attempt to refine its use in environmental epidemiological studies.

### Semen quality

Studies have inconsistently shown a decrease in semen quality in westernized countries, but have consistently shown no effect in developing countries.^22^ The decrease, if real, parallels increased rates of testicular cancer and cryptorchidism, a significant risk factor for testicular cancer.^23^ Testicular dysfunction in developed, westernized countries may be the result of multiple environmental exposures; identifying risk factors associated with geographical differences may provide causal clues. Epidemiological cohort studies could easily use this approach to monitor for subtle reproductive effects in the eastern (low exposure) and western (high exposure) areas of the Elkhorn River watershed using repeated semen quality measures that take seasonality into account. Evidence for the utility of this approach has been reported. Fertile men in Columbia, MO, had significantly lower sperm concentration and motility compared to men in New York, NY, Minneapolis, MN and Los Angeles, CA.^24^ In a nested case-control study of men with low and normal concentrations of semen, pesticide metabolite levels for alachlor and atrazine (herbicides) and diazinon (insecticide) were elevated in cases compared to controls.^25^

Work has only recently begun to evaluate the totality of HDC in the environment. Individuals are exposed to phthalates from the plastics in the environment and the water they drink, the residual anabolic steroids in the beef and dairy products they consume, and the PCBs, dioxin, TCDD, and organochlorines and other insecticide residues in food and water. Therefore, studying only one of these exposures at a time is not giving the true effect of HDCs in the environment on human health.

### Anogenital distance

With respect to reproductive insults and HDCs, a variety of markers have been studied.^26^ Some of these appear more sensitive to environmental exposures at critical developmental time points than others, although how they affect later reproductive function is unclear. For example, anogenital distance (AGD) is an antiandrogenic marker of phthalate exposure in rats.^27^,^28^ Methodologies have been developed and applied to humans for measuring AGD.^29^ A reduction in AGD was seen in male infants whose mothers were in the upper 25% of the distribution of four phthalate metabolites.^30^,^31^ AGD may be one of the best markers of subtle *in utero* changes resulting from continuous exposure to natural and xeno-hormones during embryonic development. The fetus is, without a doubt, the most vulnerable human population in the Elkhorn River watershed.

### Biomarkers of cancer risk

Increased exposure to estrogens is an established risk factor for breast cancer in epidemiological studies. Aside from reproductive risk factors, greater red meat consumption was associated with hormone positive breast cancer in 90,659 premenopausal women enrolled in the Nurses’ Health Study II and followed for 12 years.^32^ Factors that increase estradiol increase the risk for breast cancer, and clearly if drinking water contained estrogenically active compounds, these compounds may increase the probability of carcinogenesis. Exposure to estradiol has been shown to transform and initiate tumorigenesis in human breast epithelial cells.^33^,^34^ Estrogens, predominantly estrogen-3,4-quinones, react with DNA to cause mutations leading to initiation of cancer.^35^ Experiments in cultured breast cancer cells and animal models show that the formation of DNA adducts result in mutagenicity, cell transformation and carcinogenicity. The effects of some of these factors have already been observed in women with breast cancer 35,36 and men with prostate cancer,^37^ as well as several animal models for estrogen carcinogenesis.^35^ Contaminants entering streams and rivers from CAFOs can increase the formation of depurinating estrogen-DNA adducts in exposed fish, therefore, fish may act as a sensitive marker of exposure that can be used to identify areas in a watershed that put humans at greater risk of health effects.

## Conclusion

Environmental sampling is necessary for evaluating exposure to HDCs; however, sampling is not systematic in time or space, nor does it represent the time frame necessary to adequately link it to human disease outcomes. Although data from municipal sources are available and reliable, countless private drinking water wells go untested and unmonitored. These wells may be in areas vulnerable to concentrated reservoirs of contaminants due to the soil type, infiltration rate, runoff potential, organic matter and erodibility coupled with land use in the region and the chemical properties of the contaminants introduced into the environment. The lack of a defined boundary and introduction of exposure heterogeneity is one of the primary reasons why associations to health outcomes cannot be shown in environmental epidemiological studies. Greater success has been seen in occupational studies because they have natural boundaries with good denominator data, have shared and concretely defined exposures, have the ability to test intermediate hypotheses between exposure and disease, and there are other workplaces with similar exposures where the results of one study can be replicated in another.^38^ The use of the watershed addresses some of these differences between occupational and environmental epidemiological studies. The watershed provides a natural boundary and the potential within this boundary to obtain denominator data. Based on the characteristics of the watershed combined with sampling data, shared exposures can be identified and intermediate hypotheses tested using sentinel markers of exposure in fish and humans. Lastly, comparable groups identified in other watersheds with similar characteristics but different surrounding land uses can be used to replicate findings.
